# Supplementary material for: Efficient Adsorption of Ammonia by Surface-Modified Activated Carbon Fiber Mesh
Source: Nanomaterials (Basel). 2023 Oct 28;13(21):2857. doi: 10.3390/nano13212857 (PMC10648919; doi:10.3390/nano13212857)
Supplement: Supplementary file 1 [file nanomaterials-13-02857-s001.zip › nanomaterials-2653969-supplementary.pdf]

nanomaterials

Supplementary Materials

Efficient Adsorption of Ammonia by Surface-Modified Activated Carbon Fiber Mesh

Yongxiang Niu [1,2], Chao Zheng [2], Yucong Xie [2], Kai Kang [2], Hua Song [2], Shupei Bai [2], Hao Han [2,*] and Shunyi Li [1,*]

[1] School of Ecology and Environment, Zhengzhou University, Zhengzhou 450001, China; xznyxmx@163.com
[2] State Key Laboratory of NBC Protection for Civilian, Beijing 102205, China; 13263157913@163.com (C.Z.); ycong6364@163.com (Y.X.); kangkai@sklnbcpc.cn (K.K.); huasww@163.com (H.S.); baisp@263.net (S.B.)
* Correspondence: thinkinghh@163.com (H.H.); lsy76@zzu.edu.cn (S.L.)

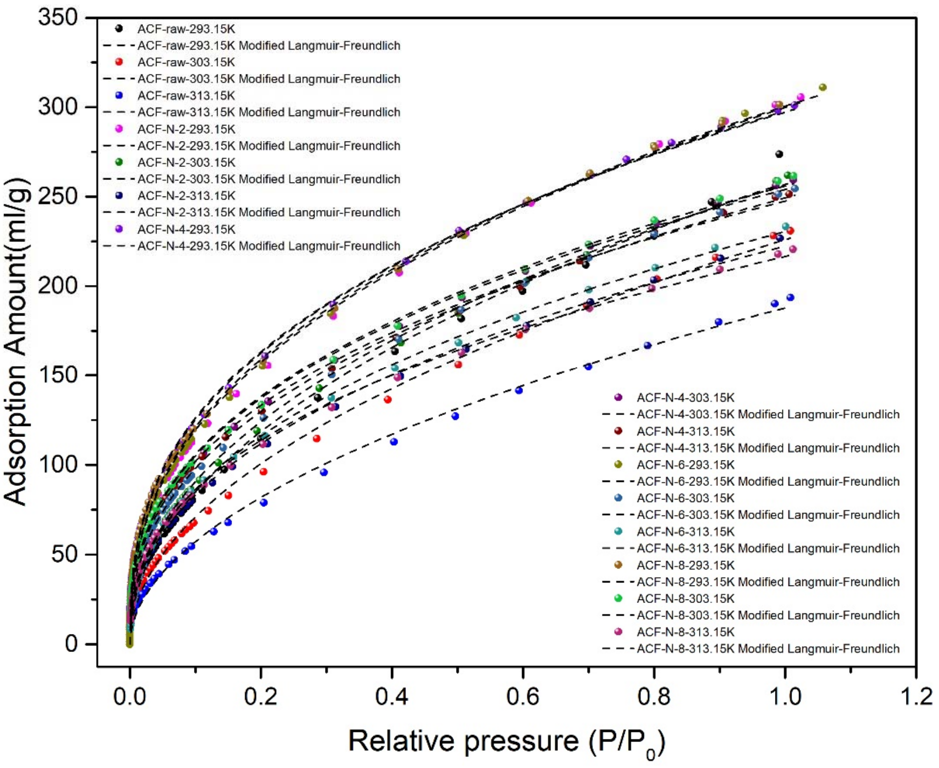

Figure S1. Ammonia adsorption isotherms of ACF fitted by Langmuir-Freundlich model.

Table S1. R-Squares of the model fit.

Adsorption isotherm	R^2
ACF-raw-293.15K	0.99576
ACF-raw-303.15K	0.99697
ACF-raw-313.15K	0.99663
ACF-N-2-293.15K	0.99806
ACF-N-2-303.15K	0.99917
ACF-N-2-313.15K	0.99898
ACF-N-4-293.15K	0.99908
ACF-N-4-303.15K	0.99912
ACF-N-4-313.15K	0.99929
ACF-N-6-293.15K	0.9989
ACF-N-6-303.15K	0.99925
ACF-N-6-313.15K	0.99942
ACF-N-8-293.15K	0.99884
ACF-N-8-303.15K	0.99902
ACF-N-8-313.15K	0.99956

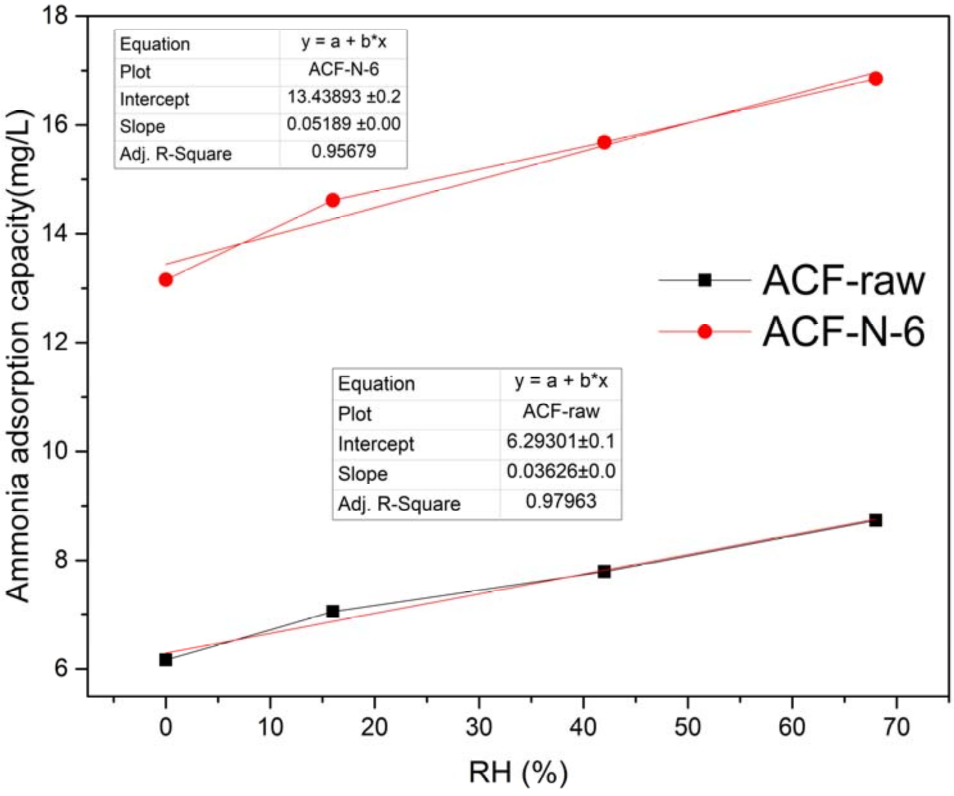

Figure S2. Correlation fitting between water content and ammonia breakthrough adsorption.

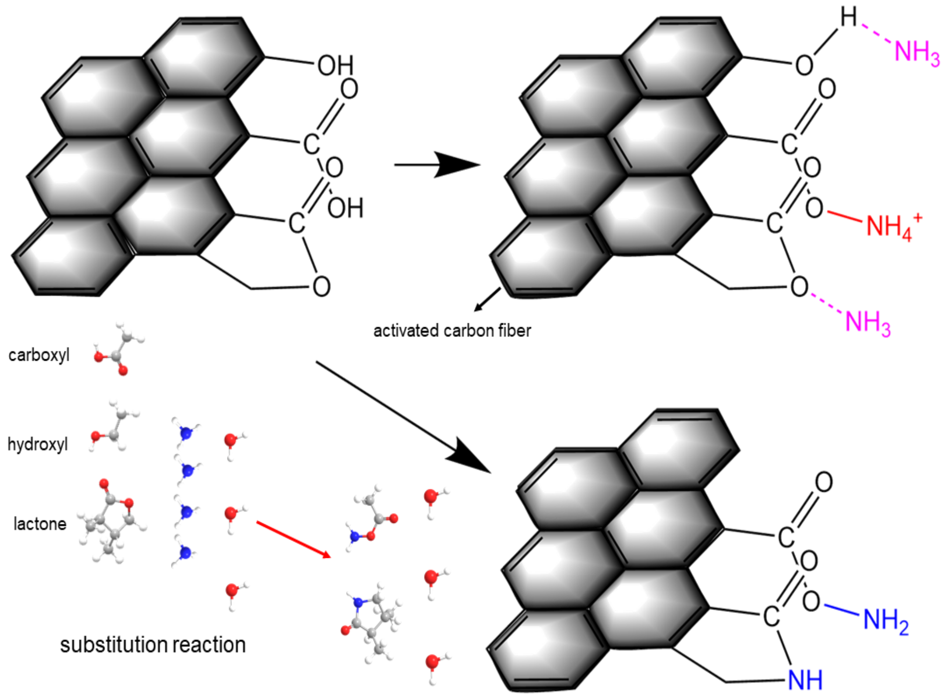

Figure S3. The binding process of ammonia with the surface functional groups of ACF.
